# Gateable Skyrmion Transport via Field-induced Potential Barrier Modulation

**DOI:** 10.1038/srep21099

**Published:** 2016-02-17

**Authors:** Hiu Tung Fook, Wei Liang Gan, Wen Siang Lew

**Affiliations:** 1 School of Physical & Mathematical Sciences, Division of Physics and Applied Physics, Nanyang Technological University, 21 Nanyang Link, Singapore 637371.

## Abstract

We report on the influence of pinning potentials on current-driven skyrmion dynamics and demonstrate that skyrmions can be gated via either magnetic or electric fields. When encountering pinning potentials, skyrmions are well known to simply skirt around them. However, we show that skyrmions can be depinned much more easily when their driving force is oriented against the pinning site rather that the intuitive option of being oriented away. This observation can be exploited together with the normally undesirable Magnus force for the creation of a skyrmion diode. The phenomenon is explained by the increased skyrmion compression resulting from the spin transfer torque opposing the repulsive potential. The smaller skyrmion size then experiences a reduced pinning potential. For practical low-power device applications, we show that the same skyrmion compression can be recreated by applying either a magnetic or electric field. Our analysis provides an insight on the skyrmion dynamics and manipulation that is critical for the realization of skyrmion-based transistors and low-power memory.

Magnetic skyrmions are topologically stable spin textures that have been found in materials with Dzyaloshinskii-Moriya (DM) interaction such as multilayers where the DM interaction arises from the broken inversion symmetry at the interfaces^1^,^2^. Since their first experimental observation at low temperatures and more recently, at room temperature^3^,^4^,^5^, there has been a growing interest in skyrmions. Due to their small size, resistance to pinning by defects and the ability to be driven under low applied current, skyrmions offer great potential as information carriers for memory devices where the existence and non-existence of skyrmions represent each of the binary bits^6^,^7^,^8^,^9^. However, there are obstacles that hinder the realization of skyrmion-based memory devices. Due to their topological stability, they are highly resistant to conventional pinning used in domain wall devices as they are known to skirt around the pinning sites. This results in a dilemma where an increased resistance to thermal fluctuations will come at the expense of requiring a high threshold current density for depinning.

Here, we demonstrate that the pinning potential and subsequently, the skyrmion motion, can be gated through voltage-controlled magnetic anisotropy (VCMA) or external magnetic field at pinning potentials introduced by adding patterned ferromagnetic layers. The ferromagnetic layers act as potential barriers due to the strong topological repulsion between the skyrmions and the ferromagnetic layers. In such systems, we found that skyrmions depin much more easily when driven against the pinning site due to a skyrmion compression mechanism. This results in an operation asymmetry where skyrmions driven in either direction experiences a significantly different pinning strength. We then show that the same compression effect can be replicated with the application of external electric or magnetic fields. By being able to modulate the pinning strength through a field-based method, the device toggles between a high thermal stability state and an efficient driving state at almost no energy cost. This unique behavior in our proposed skyrmion pinning structure is thus critical for the realization of skyrmion-based magnetic transistors and low-power skyrmion channel memory.

## Skyrmion dynamics at pinning potentials

The trajectory of current-driven skyrmion motion do not lie in the same direction as the flow of the conduction electrons. Due to the presence of the Magnus force, current-induced skyrmion motion deviates from the direction of electron flow when *α* *≠* *β.* The angle of deviation varies with the ratio of *α* to *β*^7^,^10^, where *α* is the Gilbert damping constant and *β* is the non-adiabatic constant of the spin transfer torque. The Magnus force is caused by the coupling between local magnetization and conduction electron^11^,^12^, and causes skyrmions to move at an angle from the direction of electron flow when *α* *≠* *β*. The effect of Magnus force on skyrmion motion can be understood from the equation of motion:


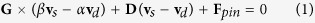


where **v**_*d*_ is the drift velocity of the skyrmion and **v**_*s*_ is the velocity of the conduction electrons. The first term on the left hand side of Equation 1 is the Magnus force, with the gyromagnetic coupling vector **G** = *G***e**_*z*_ where **e**_*z*_ is the unit vector along the *z*-direction and *G* = 4π*Q* where *Q* = −1 is the skyrmion number; the second term is the dissipative force, with **D** as the dissipative force tensor and the *D*_*ij*_ components being *D*_*xx*_ = *D*_*yy*_ = *D* and 0 otherwise; and the third term is the phenomenological pinning force (**F**_*pin*_) due to impurities, boundaries, etc. When taking **F**_*pin*_ = 0 and considering *β* ≠ *α* ≉ *0*, the additional velocity transverse to the current due to Magnus force is given by δ**v**_*d*_ = **e**_*z*_ × *G*(*α* − *β)***v**_*s*_/*D.*

As the Magnus force scales with applied current density *J*, there exists a threshold current where the Magnus force overcomes the skyrmion-edge repulsion^13^. Above this threshold current, skyrmions are annihilated at the nanowire edge, resulting in a catastrophic data loss for skyrmion-based memory devices. This can be prevented by shaping the ferromagnetic pinning layers to line the nanowire edges and act as curbs to achieve a higher repulsive force felt by skyrmions at the edge. The ferromagnetic layers can also be patterned to form pinning sites along a nanowire. This method of pinning skyrmions is advantageous when compared to the conventional pinning achieved by material removal, as it can create any arbitrarily shaped potentials while causing little disturbances to the in-plane driving current.

**Figure 1:**
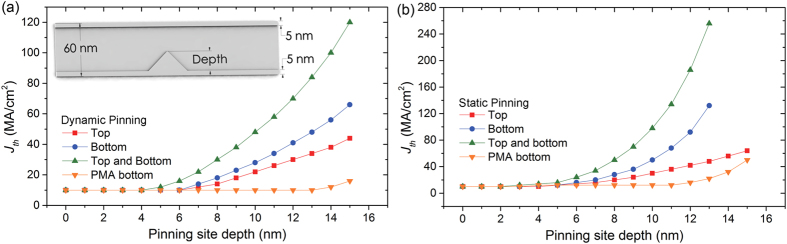
(**a**) Dynamic threshold pinning current density *J*_*th*_ as a function of pinning site depth of pinning sites at top, bottom and both edges by additional ferromagnetic layers and bottom edge by PMA patterning. Inset shows a pinning site created by an additional ferromagnetic layer at the bottom edge of the curbed nanowire, curbs are 5 nm wide and the total width of the nanowire is 60 nm. (**b**) Static threshold pinning current density *J*_*th*_ as a function of pinning site depth of pinning sites at top, bottom and both edges by additional ferromagnetic layer and bottom edge by PMA patterning. Dynamic pinning *J*_*th*_ is measured by the minimum current density required to pin a skyrmion driven from the nucleation site located 100 nm to the left of the pinning site, whereas *J*_*th*_ for static pinning was measured by the minimum current density required to depin a skyrmion that is already at the position of minimum energy. Aside from patterning the additional ferromagnetic layers to form pinning sites, the perpendicular magnetic anisotropy (PMA) of the magnetic material can also be patterned to achieve the same effect. However, the pinning strength of such pinning sites is lower.

In this section, we study the asymmetry in skyrmion dynamics induced by the Magnus force as skyrmions approach the pinning sites from opposite directions. We simulate this by driving the skyrmions in the same direction but with the pinning site placed on either the top or bottom edge of the nanowire. For the value of *β/α* = 3.5 used in our simulations, the net driving force from the spin transfer torque (STT) is oriented towards the top edge of the nanowire. As skyrmions are known to skirt around notches used in conventional domain wall pinning, a skyrmion may be expected to easily skirt around a bottom pinning site while a skyrmion driven against a top pinning site should intuitively be trapped. However, it is apparent from Fig. 1 that the opposite is true, i.e., a skyrmion driven directly against the top pinning site due to Magnus force has a lower *J*_*th*_.

Two types of pinning were also studied, a static case where the skyrmion is nucleated beside the pinning site and also a dynamic case where a skyrmion is driven to its terminal velocity before allowed to interact with the pinning site. The presence of a topological inertia in skyrmionic spin textures predicts that a moving skyrmion will possess additional energy^14^. Indeed, it was found that skyrmions in the dynamic case could depin using half the current densities required in static case, as shown in Fig. 1a,b. Our results here highlight that it is important to take into consideration the expected skyrmion speed, for the design of pinning sites.

**Figure 2:**
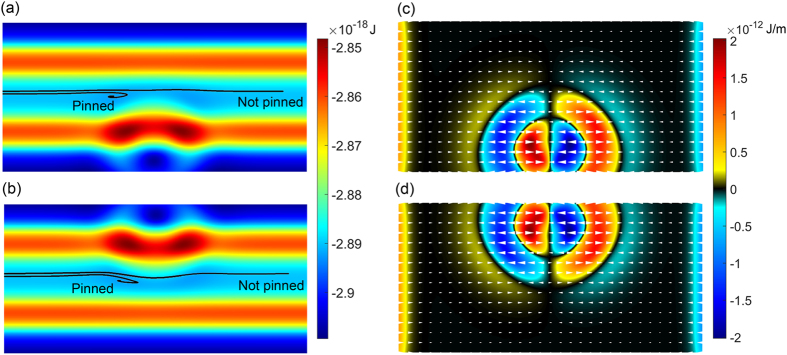
(**a**) 2D plot of total energy of the system as a function of skyrmion position in a nanowire with a potential barrier at the bottom edge. Black solid lines are trajectories of skyrmions driven from left to right by low current density pinned at the pinning potential and driven by high current density not pinned by the pinning potential. (**b**) 2D energy plot for a nanowire with a pinning barrier at the top edge (**c**) 2D plot of force acting in the positive *x*-direction on a skyrmion travelling in the *x*-direction as a function of skyrmion position in a nanowire with a ferromagnetic pinning layer at the bottom edge. The white arrows show the direction of force acting on the skyrmion and scales with the strength, forces on the left and right edge originate from skyrmion-edge repulsion. (**d**) 2D force plot for nanowire with a ferromagnetic pinning layer at the top edge.

Figure 2a,b shows the energy landscape experienced by a skyrmion in a nanowire with pinning sites, with the trajectories of a pinned and unpinned skyrmion superimposed. When driven at *J* < *J*_*th*_, a skyrmion is pinned at the pinning potential and stays at a position of lowest energy before the pinning site. In the unpinned case, the skyrmion is driven at a higher current *J* > *J*_*th*_, the trajectory of a skyrmion is closer to the top nanowire edge due to higher Magnus force. When a skyrmion is driven against a ferromagnetic pinning layer, as is the case in Fig. 2b, the skyrmion is compressed to the required size for depinning. The compression arises from the STT and the opposing topological repulsion from the inner edges of the ferromagnetic pinning layer^15^,^16^. In the case of a pinning site with ferromagnetic layers at both edges of the nanowire, a higher *J*_*th*_ is required for depinning compared to pinning layers at only one edge since the total pinning depth is effectively doubled, as illustrated in Figure S2. However, for the top and bottom pinning site configuration, the *J*_*th*_ for a skyrmion passing in both direction is constant. Such a symmetric behavior is more suitable for memory devices as it is expected that the data will be translated back and forth equally.

Two main types of forces act on a skyrmion near a ferromagnetic barrier. Firstly, it experiences a weak long range attractive force depicted by the light yellow region and a strong short range repulsive force depicted by the dark blue region in Fig. 2c,d. The long range attractive force results from the demagnetizing field emanating from the ferromagnetic barrier which favors the magnetization of the skyrmion^17^. On the other hand, the short range repulsive force originates from the skyrmion’s magnetization overlapping into the ferromagnetic barrier when the skyrmion edge is in close proximity to the barrier. Therefore, a skyrmion approaching a pinning site will first accelerate then decelerate. If the repulsive force is stronger than the driving force, the skyrmion decelerates to a stop and becomes pinned. An increase in the pinning site depth will decrease the gap available for the skyrmion to traverse without experiencing repulsion from the ferromagnetic barrier. Animated movies showing the described skyrmion motion are included in Supplementary Movies S4,S5,S6 to S7.

**Figure 3:**
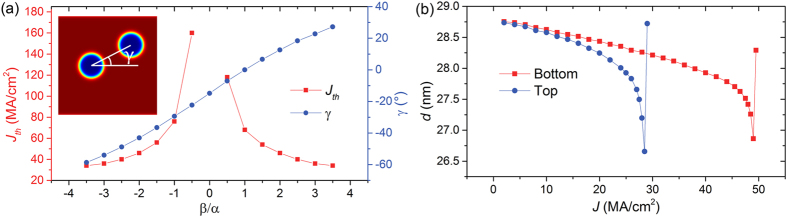
(**a**) Threshold current density *J*_*th*_ needed to depin a skyrmion pinned by a pinning site of depth = 10 nm located at the top edge of the nanowire and the angle of deviation *γ* of the skyrmion motion to the direction of electron flow as a function of the ratio of non-adiabatic constant of the STT *β* to the Gilbert damping constant *α.* At *β* = 0, pinned skyrmions are not depinned due to the lack of a driving force. Only *β* is varied while *α* is kept at a constant value of 0.1. Inset shows the deviation angle γ measured from the center of the skyrmion before and after driving by current. (**b**) Skyrmion diameter *d* as a function of applied current density *J* for ferromagnetic pinning layers located at opposite edges of the nanowire.

The effect of Magnus force can be analyzed by considering four general cases of *β* as shown in Fig. 3a. When *β* > *α*, the skyrmion moves with a positive deviation angle towards the top edge of the magnetic film. Conversely, *β* < *α* results in a negative deviation. The deviation angle is zero when *β* = *α*, i.e. the Magnus force is non-existent. At *β* = 0, skyrmions are not depinned even when high current densities are applied due to the lack of a driving force as expected from Equation 1. As the magnitude of *β* increases, *J*_*th*_ is lowered due to a higher current-induced skyrmion driving force. For positive *γ*, a lower *J* is needed for the skyrmion to reach the threshold size *d*_*th*_ as the skyrmion is compressed further when driven against the pinning site at the top nanowire edge. For negative *γ*, the skyrmion is not compressed as significantly because the Magnus force drives it away from the pinning site, thus a higher *J*_*th*_ is observed. This skyrmion transport asymmetry can therefore be exploited for a skyrmion diode, where skyrmion transport in different directions have a significantly different *J*_*th.*_

To determine *d*_*th*_ and verify the effect of the direction of skyrmion motion on *J*_*th*_, *β* and *α* are set to constant values of 0.35 and 0.1 respectively. *J*_*th*_ for pinning sites at opposite edges of the nanowire is compared in Fig. 3b, a pinning site at the top edge of the nanowire is more efficient at compressing the skyrmion. As such, a lower *J* is required to compress the skyrmion to *d*_*th*_ of ~26.8 nm in the case of a 10 nm deep pinning site. The asymmetric pinning behavior is general for skyrmions in a nanowire; asymmetry is always observed, regardless of pinning site geometry. Figure S1 in Supplementary Information shows that the asymmetry remains even with cuboidal or cylindrical pinning sites.

To achieve operation symmetry in the presence of Magnus force, we use a symmetric pinning site by adding ferromagnetic layers on both the top and bottom edges of the nanowire. Shown in Figure S2, the repulsive and attractive forces acting on the skyrmion by the pinning site are larger than that of a single pinning site. The acceleration and deceleration of the skyrmion is therefore more prominent as shown in Supplementary Movies S8 and S9. The larger repulsive force also translate to a higher barrier potential experienced by the pinned skyrmion, when compared to the case of a single pinning site, as shown in Figure S2. An increase in pinning site depth results in a reduction of the gap width that the skyrmion can traverse pass without experiencing the strong topological repulsion. A larger *J* is thus required to drive and compress the pinned skyrmion to the threshold size in order to pass through the allowance at the center region.

**Figure 4:**
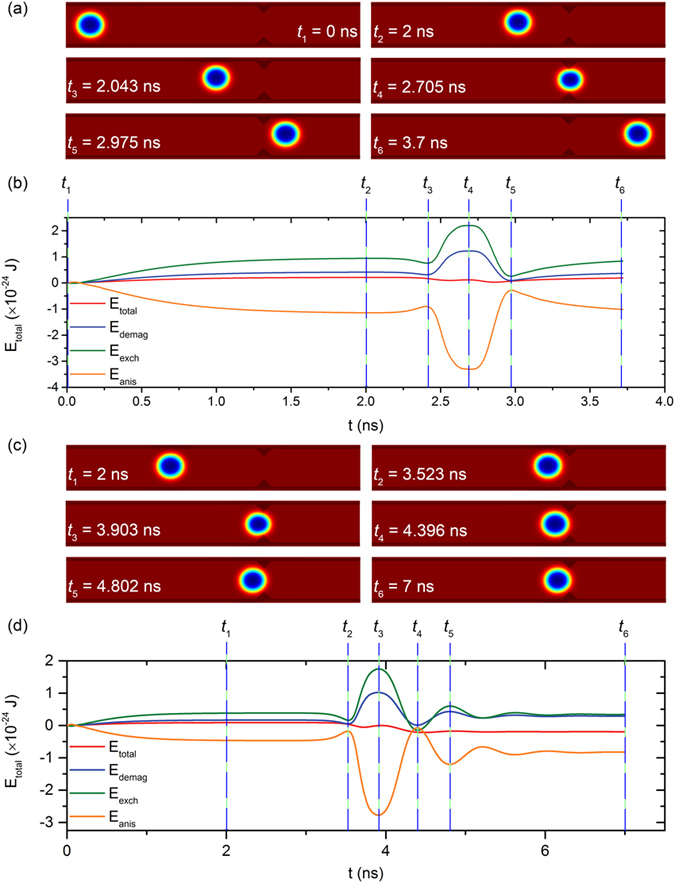
Snapshots of the micromagnetic simulation at different times of a skyrmion (**a**) passing through and (**c**) pinned by a symmetric pinning site. The time-trace of demagnetizing, exchange, anisotropy and total energy of the system of both cases are shown in (**b**) and (**d**) respectively. Vertical dash lines represent the times corresponding to the simulation snapshots.

The skyrmion dynamics at symmetric pinning sites can be understood by analyzing the energy changes in the system. The demagnetizing, anisotropy, exchange as well as the total energy were calculated and plotted as a function of time in Fig. 4a. Starting from a relaxed state, snapshots of the magnetization at different times are shown in Fig. 4b. When a driving current is applied, the skyrmion is driven against the top edge of the nanowire due to Magnus force, resulting in a slightly compressed and elliptical skyrmion. The compression is evident in the decrease of the anisotropy energy while the ellipticity results in an increase of the exchange energy, as the elliptical shape has a larger perimeter per area ratio. However, the different energy changes do not completely cancel each other out, as the compressed state is of a higher energy than a skyrmion in its remnant state. At *t*_*3*,_ the skyrmion begins to enter a potential well induced by the weak long range attraction from the pinning site’s demagnetizing field. Consequently, a slight decrease in the demagnetizing energy *E*_*demag*_ of the system is observed. At *t*_*4*_, the skyrmion is sandwiched between the two pinning sites and its size is at a minimum. After this time, the skyrmion has successfully depinned; even if the driving current is removed, it will not return to the left of the pinning site. At *t*_*5*_, the skyrmion has successfully escaped the potential well on right of the pinning site and can travel unimpeded. In the other case where *J* < *J*_*th*_, the skyrmion never crosses the midway point of the pinning site. To dissipate its kinetic energy, it repeatedly “bounces” when driven against the pinning site. After 4 ns, the skyrmion motion is damped to a complete stop. Depinning is now impossible unless a much higher static *J*_*th*_ is supplied. The abovementioned dynamics are shown in Fig. 4c,d.

### Field-induced skyrmion compression

In our analysis thus far, we have established that the topological repulsion and its resulting skyrmion compression is the single most important factor influencing the magnitude of *J*_*th*_. While skyrmions can be compressed by spin transfer torque, it is highly inefficient. For practical device applications, we present efficient methods to achieve the same compression, either by applying an external magnetic field *B*_*z*_ or by VCMA. To understand the effect of an external magnetic field on a skyrmion, we first model the skyrmion cross section using a 360° domain wall profile^18^,^19^,^20^,^21^,


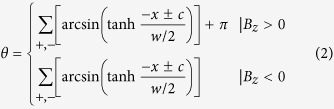


where *θ* is the polar angle of the magnetization at position *x*, *c* and *w* are the position and width of two overlapping 180° domain walls respectively. The diameter of the skyrmion is twice the width *w* and the field-dependent values of *c* and *w* are given by,


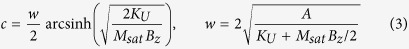


where *K*_*U*_ is the PMA value, *M*_*sat*_ is the saturation magnetization, *B*_*z*_ is the external magnetic field in the z-direction and *A* is the exchange stiffness. The diameter of the skyrmion roughly scales with 1/(*B* − *B*_*0*_) as numerically calculated^18^,^21^,^22^, where *B*_*0*_ is the critical field at which the skyrmion diameter goes to infinity. From Equation 3, it is evident that a change in PMA value *K*_*U*_has a similar effect to *B*_*z*_ on the skyrmion size. The change in *K*_*U*_ can be effected by applying an electric field. Due to the spin-dependent screening of the applied electric field, magnetic anisotropy at the surface of the ferromagnet is varied. The effect can be varied locally due to charge accumulation at the surface and scales linearly with the applied electric-field strength^23^,^24^,^25^,^26^. The temporal evolution of skyrmions in thin films of different geometries, including a curbed nanowire structure identical to that used in our proposed structure, were studied in response to a time-varying *K*_*U*_ and *B*_*z*_. When the external stimuli are applied as a series of Heaviside step function, skyrmion breathing modes were observed to be excited. The modal frequencies were found to be heavily dependent on skyrmion size and thin film geometry, with smaller skyrmion sizes and restrictive geometries generally exhibiting higher frequency breathing modes. Although the skyrmion breathing is manifested as oscillatory expansions and compressions of the skyrmion, no significant changes in *J*_*th*_ could be attributed to the skyrmion breathing. This may be explained by the high frequencies of the breathing (~9 GHz), which does not allow the compressed skyrmion enough time to overcome the gap before expanding again.

### Gating of skyrmions via potential barrier modulation

**Figure 5:**
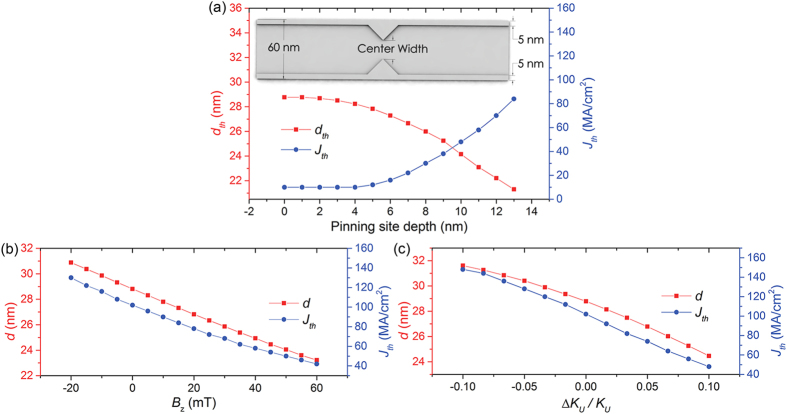
(**a**) Threshold diameter of skyrmion *d*_*th*_ passing through the pinning site and threshold current density *J*_*th*_ as a function of pinning site depth. The inset shows the width of the center region which is reduced when pinning site depth is increased. (**b**) Red plot shows the equilibrium diameter of skyrmion *d* in a curbed nanowire at the located away from the pinning potential. Blue plot shows the static threshold current density *J*_*th*_ for a ferromagnetic pinning layer with depth = 10 nm as a function of external magnetic field *B*_*z*_ applied in the z-axis direction (**c**) and as a function of the voltage-induced PMA change Δ*K*_*U*_/*K*_*U*_, where *K*_*U*_ = 6×10^5^ J/m^3^.

Using an external magnetic field to reduce the skyrmion size will reduce the topological repulsion the skyrmion experiences when it is near to a pinning site. *J*_*th*_ is therefore also expected to be reduced. This effect may be used in a skyrmion channel memory where the pinning strength can be modulated. Shown in Fig. 5b, *J*_*th*_ is reduced by 60% when an external magnetic field of 60 mT is applied to a pinning site with a depth of 10 nm. When the static *J*_*th*_ is lowered to below the dynamic *J*_*th*_, current-driven skyrmions are not pinned regardless of its initial velocity. In the demonstrated skyrmion channel memory, pinning sites are separated by a distance larger than the threshold to avoid repulsion between skyrmions that causes inconsistent *J*_*th*_^17^. The driving current density *J* is kept at a constant value of 45 MA/cm^2^.

**Figure 6:**
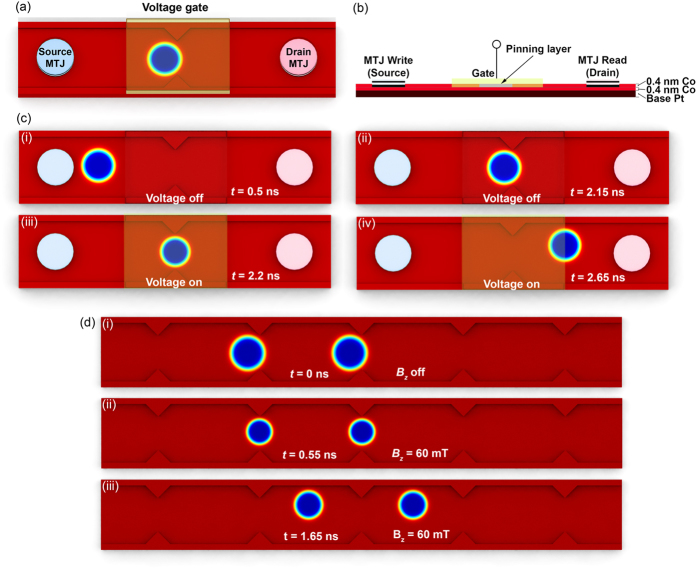
(**a**) Design of the skyrmion transistor with MTJs for nucleating and sensing skyrmions and a voltage-gated region around the artificial pinning site. (**b**) Cross-section diagram of the skyrmion transistor cut along the middle. (**c**) Snapshots of the skyrmion transistor operation. At times *t* = 0.5 ns and *t* = 2.15 ns corresponding to (i) and (ii) respectively, the skyrmion is pinned at the pinning site due to topological repulsion from the pinning site. When a gate voltage is applied, VCMA causes the skyrmion to decrease in size resulting in lowered topological repulsion. The compressed skyrmion depins at time *t* = 2.2 ns and travels to the opposite end of the nanowire at *t* = 2.65 ns, corresponding to (iii) and (iv). (**d**) Snapshots of the skyrmion memory operation. Pinning sites are at fixed separation distances to prevent skyrmion to skyrmion interaction. (i) Skyrmions are initially pinned at time *t* = 0 ns. When an out-of-plane magnetic field of strength 60 mT is applied, they are compressed to a smaller size resulting in lower topological repulsion experienced. The compressed skyrmions can then depin and traverse down the memory line without getting pinned as seen at times (ii) *t* = 0.55 ns and (iii) *t* = 1.65 ns until the magnetic field is switched off. Skyrmions can thus be driven when an external out-of-plane magnetic field is applied and pinned in its absence.

Other than applying a magnetic field, VCMA can also be utilized to decrease the skyrmion size. As *K*_*U*_ varies linearly with an electric field applied perpendicular to the film plane^24^,^25^,^26^, The size of skyrmions can be controlled by a voltage-gated region in the nanowire. A transistor-like behavior is observed when altering the skyrmion size by voltage-induced control of *K*_*U*_ as shown in Fig. 6. A transistor “off” state is observed when the electric field is off, i.e., the skyrmion is pinned at the pinning site. Correspondingly, when the PMA is varied to 1.025 *K*_*U*_ by the electric field, an “on” state is observed. Such a change can be generated when a gate voltage of about 400 mV is applied to FePt. CoPt films are more susceptible to electric field effects and a 2 nm thick film would show a 9% change in magnetic anisotropy energy^27^. The change in PMA would be even larger for ultrathin films such as that used in our simulations. A positive voltage induced PMA change decreases the size of a skyrmion in the region for easy depinning. Therefore, the presence of an applied voltage corresponds to the transistor “on” and “off” states and its operation is shown in Fig. 6c.

By patterning regularly spaced skyrmion gates, a periodic potential for skyrmions can be generated for a skyrmion channel memory. In this device, an external magnetic field is utilized to toggle the pinning strength of the skyrmion gates. Pinning strength can be switched between low and high for the purpose of skyrmion driving (writing/reading information) or thermal stability (data storage). The design and operation of the skyrmion channel memory is shown in Fig. 6d. In the low pinning state, skyrmions can be easily shifted between adjacent potential wells while in the high pinning state, the skyrmion is returned to its original size or even enlarged, offering extra protection against thermal fluctuations. Furthermore, the ferromagnetic layers also produce a demagnetizing field in the direction of the skyrmion core that stabilizes the skyrmion. The operation of the transistor in the ‘off’ and ‘on’ states and the skyrmion channel memory are shown in Supplementary Movies S1,S2, to S3.

In summary, we have investigated the asymmetric behavior of skyrmions at pinning sites and proposed several potential applications for the phenomena. We show that strong potential barriers can be formed by patterning additional ferromagnetic layers. By patterning the layers as pinning sites or guiding tracks, the usually undesirable Magnus force can be either utilized as a skyrmion diode or negated for operation symmetry. In the proposed skyrmion diode, a skyrmion being driving in different direction requires a significantly different *J*_*th*_, thereby allowing skyrmion flow in only 1 direction. The skyrmion depinning mechanism was analyzed and it was found that the topological repulsion and its resulting skyrmion compression determines the threshold depinning current density *J*_*th*_. The skyrmion compression arises from the STT that opposes the topological repulsion from the pinning layer. We demonstrate that the compressive effect can be replicated by applying magnetic or electric fields without the need to increase the driving current. This allows for the low current gateable skyrmion transport that is critical for the operation of the presented skyrmion transistor and skyrmion channel memory.

## Methods

Micromagnetic simulations were performed using MuMax3^28^,^29^. The magnetization dynamics of skyrmions driven by in-plane current can be modelled by the modified Landau-Lifshitz-Gilbert (LLG) equation^30^,^31^,^32^.


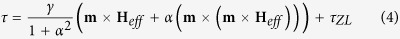



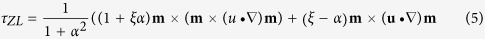



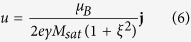


where *γ* is the electron gyromagnetic ratio, **m** is the normalized unit vector of the local magnetization, **H**_*eff*_ is the effective field, *α* is the Gilbert damping constant, *e* is the elementary charge, *M*_*sat*_ is the saturation magnetization, **j** is the current density vector and *ξ* is the degree of the non-adiabacity. A detailed derivation of the corrected Thiele equation shown in Equation 1 from the modified LLG equation can be found in Supplementary Information.

The material parameters used in the simulations corresponds to that of Co/Pt multilayers as shown in Table 1^33^,^34^. The nanowire thickness is *t*_*nanowire*_ = 0.4 nm and the width is *w*_*nanowire*_ = 60 nm. The width of the curbs are *w*_*curb*_ = 5 nm, with a thickness of *t*_*curb*_ = 0.4 nm. The patterned curb layers act as pinning layers. Pinning sites have a 90° edge pointing inward of the nanowire. The cell size used in the simulations is 1 × 1 × 0.4 nm^3^.

## Additional Information

**How to cite this article**: Fook, H. T. *et al.* Gateable Skyrmion Transport via Field-induced Potential Barrier Modulation. *Sci. Rep.* **6**, 21099; doi: 10.1038/srep21099 (2016).

## Supplementary Material

Supplementary Information

Supplementary movie 1

Supplementary movie 2

Supplementary movie 3

Supplementary movie 4

Supplementary movie 5

Supplementary movie 6

Supplementary movie 7

Supplementary movie 8

Supplementary movie 9

## Figures and Tables

**Table 1:** Material parameters used in micromagnetic simulations corresponding to cobalt/platinum multilayers.

Symbol	Quantity	Value
*M*_*sat*_	Saturation magnetization	580 × 10^3^ A/m
*K*_*U*_	Uniaxial anisotropy constant	6 × 10^5^ J/m^3^
*D*	Dzyaloshinskii-Moriya interaction strength	3 × 10^−3^ J/m^2^
*A*	Exchange stiffness	15 × 10^−12^ J/m
*β*	STT non-adiabatic constant	0.35
*α*	Gilbert damping	0.1
	constant	
*P*	Spin polarization	0.7
