# Supplementary material for: Gateable Skyrmion Transport via Field-induced Potential Barrier Modulation
Source: Sci Rep. 2016 Feb 17;6:21099. doi: 10.1038/srep21099 (PMC4756321; doi:10.1038/srep21099)
Supplement: Supplementary Information [file srep21099-s1.doc]

**Gateable Skyrmion Transport via Field-induced Potential Barrier Modulation**

**Hiu Tung Fook**^1^**, Wei Liang Gan**^1^**, and Wen Siang Lew***

^1^ School of Physical & Mathematical Sciences, Division of Physics and Applied Physics, Nanyang Technological University, 21 Nanyang Link, Singapore 637371

**SUPPLEMENTARY INFORMATION**

**Supplementary Movie 1**. Skyrmion transistor at "off" state in the absence of applied gate voltage.

**Supplementary Movie 2.** Skyrmion transistor at "on" state in the presence of applied gate voltage.

**Supplementary Movie 3.** Skyrmion memory operation with initial "off" state. External out-of-plane magnetic field switches on and off at times *t* = 2 ns and *t* = 4.84 ns respectively.

**Supplementary Movie 4.** Simulation of a skyrmion driven towards a pinning site located at the bottom nanowire edge by current density below that of threshold depinning. The skyrmion is thus pinned by the pinning site.

**Supplementary Movie 5.** Simulation of a skyrmion driven towards a pinning site located at the bottom nanowire edge by current density above that of threshold depinning. The skyrmion is not pinned by the pinning site.

**Supplementary Movie 6.** Simulation of a skyrmion driven towards a pinning site located at the top nanowire edge by current density below that of threshold depinning. The skyrmion is thus pinned by the pinning site.

**Supplementary Movie 7.** Simulation of a skyrmion driven towards a pinning site located at the top nanowire edge by current density above that of threshold depinning. The skyrmion is not pinned by the pinning site.

**Supplementary Movie 8.** Simulation of a skyrmion driven towards a symmetric pinning site by current density below that of threshold depinning. The skyrmion is thus pinned by the pinning site.

**Supplementary Movie 9.** Simulation of a skyrmion driven towards a symmetric pinning site by current density above that of threshold depinning. The skyrmion is not pinned by the pinning site.

**Skyrmion Equation of Motion**

We start our discussion by understanding the skyrmion motion as derived from the basic magnetization dynamic equation. Under the application of current, the magnetization dynamics is expressed by the modified Landau-Lifshitz-Gilbert equation, which includes the adiabatic and the non-adiabatic contribution from the spin-transfer torque (STT).

$0=\frac{\alpha}{\gamma M_{s}^{2}}\mathbf{M}\times\frac{d\mathbf{M}}{dt}-\frac{1}{\gamma M_{s}}\frac{d\mathbf{M}}{dt}-\frac{1}{M_{s}}\mathbf{M}\times\mathbf{H}_{eff}-\frac{1}{\gamma M_{s}^{2}}\left(\mathbf{u}\cdot\nabla\right)\mathbf{M}+\frac{\beta}{\gamma M_{s}^{2}}\mathbf{M}\times\left(\left(\mathbf{u}\cdot\nabla\right)\mathbf{M}\right)$ (1),

where **M** is the magnetization, *M­_s_* is the saturation magnetization, *γ* is the gyromagnetic ratio, **H***_eff_* is the effective field, **u** is the electron drift velocity, *α* is the Gilbert damping factor and *β* is the non-adiabaticity of the STT.

By dividing the torque terms by *M_s_* and *γ*, we obtain the effective fields:

$0=\mathbf{H}_{g}+\mathbf{H}_{d}+\mathbf{H}_{\mathrm{int}}+\mathbf{H}_{adia}+\mathbf{H}_{nonadia}$ (2),

where *H_g_*, *H­_d_*, *H_int_, H_adia_, H_nonadia_* represent the gyroscopic field, the dissipative field, the internal field, the adiabatic spin torque field, and the nonadiabatic spin torque field respectively. The fields are then changed to forces by performing the following mathematical operation:

$\mathbf{F}=\int\mathbf{f}dV=-\int\nabla_{M}\left(H\cdot M\right)dV$ (3),

which turns the LLG equation into a force equilibrium equation:

$0=\mathbf{F}_{g}+\mathbf{F}_{d}+\mathbf{F}_{\mathrm{int}}$ (4),

where *F_g_*, *F_d_*, and *F_int_* correspond to the gyroscopic force, dissipative force, and the internal force, respectively. In equilibrium, the skyrmion moves with a constant velocity *v*, and the whole system can be regarded as a wave the travel at the same velocity.

$M=M\left(x-vt\right)$ (5),

which also gives us

$\frac{d\mathbf{M}}{dt}=-\left(\mathbf{v}\cdot\nabla\right)\mathbf{M}$ (6).

The relation between *d****M****/dt* and *v* allows us to re-express the force equation, which will yield the skyrmion motion equation. For instance, the gyroscopic force is expressed as:

$\left(\begin{matrix} F_{x} \\ F_{y} \\ F_{z} \end{matrix}\right)_{g}=-\left(\begin{matrix} H_{x}\frac{\partial M_{x}}{\partial x}+H_{y}\frac{\partial M_{y}}{\partial x}+H_{z}\frac{\partial M_{z}}{\partial x} \\ H_{x}\frac{\partial M_{x}}{\partial y}+H_{z}\frac{\partial M_{z}}{\partial y}+H_{y}\frac{\partial M_{y}}{\partial y} \\ H_{x}\frac{\partial M_{x}}{\partial z}+H_{y}\frac{\partial M_{y}}{\partial z}+H_{z}\frac{\partial M_{z}}{\partial z} \end{matrix}\right)_{g}=-\left(\begin{matrix} \frac{\partial M_{x}}{\partial x} & \frac{\partial M_{y}}{\partial x} & \frac{\partial M_{z}}{\partial x} \\ \frac{\partial M_{x}}{\partial y} & \frac{\partial M_{z}}{\partial y} & \frac{\partial M_{y}}{\partial y} \\ \frac{\partial M_{x}}{\partial z} & \frac{\partial M_{y}}{\partial z} & \frac{\partial M_{z}}{\partial z} \end{matrix}\right)\left(\begin{matrix} H_{x} \\ H_{y} \\ H_{z} \end{matrix}\right)_{g}$ (7).

Substituting the expression for the gyro field (*H_g_*):

$\mathbf{H}_{g}=\left(\begin{matrix} H_{x} \\ H_{y} \\ H_{z} \end{matrix}\right)_{gyro}=\frac{\alpha}{M_{s}}\mathbf{M}\times\frac{d\mathbf{M}}{dt}=\frac{\alpha}{\gamma M_{s}^{2}}\left(\begin{matrix} M_{y}\frac{dM_{z}}{dt}-M_{z}\frac{dM_{y}}{dt} \\ M_{z}\frac{dM_{x}}{dt}-M_{x}\frac{dM_{z}}{dt} \\ M_{x}\frac{dM_{y}}{dt}-M_{y}\frac{dM_{x}}{dt} \end{matrix}\right)$ (8).

Which gives us the following:

$\left(f_{i}\right)_{gyro}=\alpha\left(g_{ji}v_{j}+g_{ki}v_{k}\right)$ (9),

Where $g_{ji}$is given by:

$g_{ji}=\frac{1}{\gamma M_{s}^{2}}\mathbf{M}\cdot\left(\frac{\partial\mathbf{M}}{\partial j}\times\frac{\partial\mathbf{M}}{\partial i}\right)$ (10).

The force density can then be expressed as:

$\mathbf{f}_{gyro}=\alpha\left[\begin{matrix} 0 & -g_{xy} & g_{zx} \\ g_{xy} & 0 & -g_{yz} \\ -g_{zx} & g_{yz} & 0 \end{matrix}\right]\left[\begin{matrix} v_{x} \\ v_{y} \\ v_{z} \end{matrix}\right]=\alpha\left[\begin{matrix} 0 & -g_{Z} & g_{Y} \\ g_{Z} & 0 & -g_{X} \\ -g_{Y} & g_{X} & 0 \end{matrix}\right]\left[\begin{matrix} v_{x} \\ v_{y} \\ v_{z} \end{matrix}\right]$ (11).

In vector form, this can be written as

$\mathbf{f}_{gyro}=\mathbf{g}\times\alpha\mathbf{v}$ (12),

The total gyro force is then equal to

$\mathbf{F}_{gyro}=\int\mathbf{f}_{gyro}dV=\mathbf{G}\times\mathbf{v}=\left(\int\mathbf{g}_{x}+\mathbf{g}_{y}+\mathbf{g}_{z}dV\right)\times\alpha\mathbf{v}=4\pi\hat{\mathbf{z}}\times\alpha\mathbf{v}$ (13).

Similar treatment is given to the dissipative force. The dissipative field is given by:

$\mathbf{H}_{d}=-\frac{1}{\gamma M_{s}}\frac{d\mathbf{M}}{dt}$ (14).

If we substitute $\frac{d\mathbf{M}}{dt}$:

$d_{ij}=-\left(\frac{\partial Mx}{\partial i}\frac{\partial M_{x}}{\partial j}+\frac{\partial My}{\partial i}\frac{\partial M_{y}}{\partial j}+\frac{\partial Mz}{\partial i}\frac{\partial M_{z}}{\partial j}\right)$ (15).

However we see that

$\int_{i\neq j} d_{ij}dV=0$ (16).

And thus the dissipative force can be simplified as

$\mathbf{F}_{d}=\int\mathbf{f}_{d}dV=\frac{1}{\gamma M_{s}}\left(\begin{matrix} d_{xx} & 0 & 0 \\ 0 & d_{yy} & 0 \\ 0 & 0 & d_{zz} \end{matrix}\right)\left(\begin{matrix} v_{x} \\ v_{y} \\ v_{z} \end{matrix}\right)=\mathbf{D}\cdot\mathbf{v}$ (17).

For the effective internal field, the corresponding force is considered as 0 when no external magnetic field is applied.

$F_{in}=\int\nabla_{M}\left(\mathbf{H}_{\mathrm{int}}\cdot\mathbf{M}\right)dV=0$ (18).

For the adiabatic spin torque field, we see its expression is very much similar to the dissipative field after the substitution of dM/dt=-v∙∇M:

$\mathbf{H}_{adia}=-\frac{1}{\gamma M_{s}}\left(\mathbf{u}\cdot\nabla\right)\mathbf{M}$ (19),

but with negative sign and different speed vector (*u* instead of *v*). Hence, we can immediately deduce that the adiabatic force can be expressed by:

$\mathbf{F}_{adia}=-\mathbf{D}\cdot\mathbf{u}$ (20).

For the nonadiabatic spin torque field, it is expressed by:

$\mathbf{H}_{nonadia}=\frac{\beta}{\gamma M_{s}^{2}}\mathbf{M}\times\left(\left(\mathbf{u}\cdot\nabla\right)\mathbf{M}\right)$ (21),

Similarly, we see that it is very similar to the gyroscopic field, but with different sign and constant. Hence, we can immediately deduce that the non-adiabatic force takes the form of

$\mathbf{F}_{nonadia}=-\mathbf{G}\times\beta\mathbf{u}$ (22).

The force equation, which is also known as the Thiele equation, now becomes:

$0=\mathbf{G}\times\left(\alpha\mathbf{v}-\beta\mathbf{u}\right)+D\cdot\left(\mathbf{v}-\mathbf{u}\right)$ (23).

Which shows that the skyrmion speed (*v*) can be directly related to the electron drift velocity (*u*) by:

$v_{x}=\left(\frac{\beta}{\alpha}+\frac{\alpha-\beta}{\alpha^{3}\left(G/D\right)^{2}+\alpha}\right)u$ (24),

$$v_{y}=\left(\frac{\left(\beta-\alpha\right)\left(G/D\right)}{\alpha^{2}\left(G/D\right)^{2}+1}\right)u$$

**Skyrmion Operation Asymmetry at Pinning Site**

The skyrmion operation asymmetry is a general phenomenon regardless of pinning site geometry as the topological repulsion between skyrmions and pinning sites is always present. Other than the triangular pinning sites discussed in the main text, the operation asymmetry can also be observed in cuboidal and cylindrical pinning sites. Therefore, these type of geometries can also be used for a skyrmion diode.

**
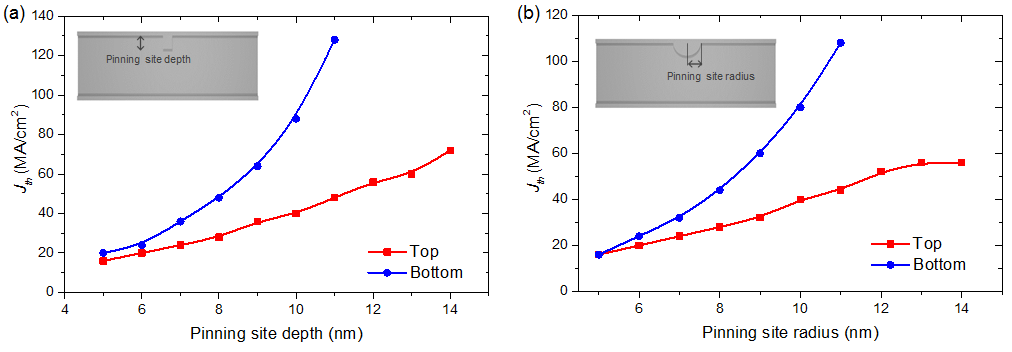
**

**Supplementary Figure S1.** Threshold depinning current density as a function of pinning site depth for a pinning site located at the top (red) and at the bottom (blue) of the nanowire, for a (a) cuboidal pinning site and (b) a cylindrical pinning site.

**Skyrmion at a Symmetrical Pinning Site**

The energy landscape experienced by the skyrmion in a symmetrical double pinning site is shown in Figure S2. As compared to a single pinning site, the potential barrier is doubled as the skyrmion has to traverse through a gap that is halved in size. The weak long range interaction also becomes a superposition of two such interactions from the top and bottom pinning sites, resulting in a much stronger potential well.


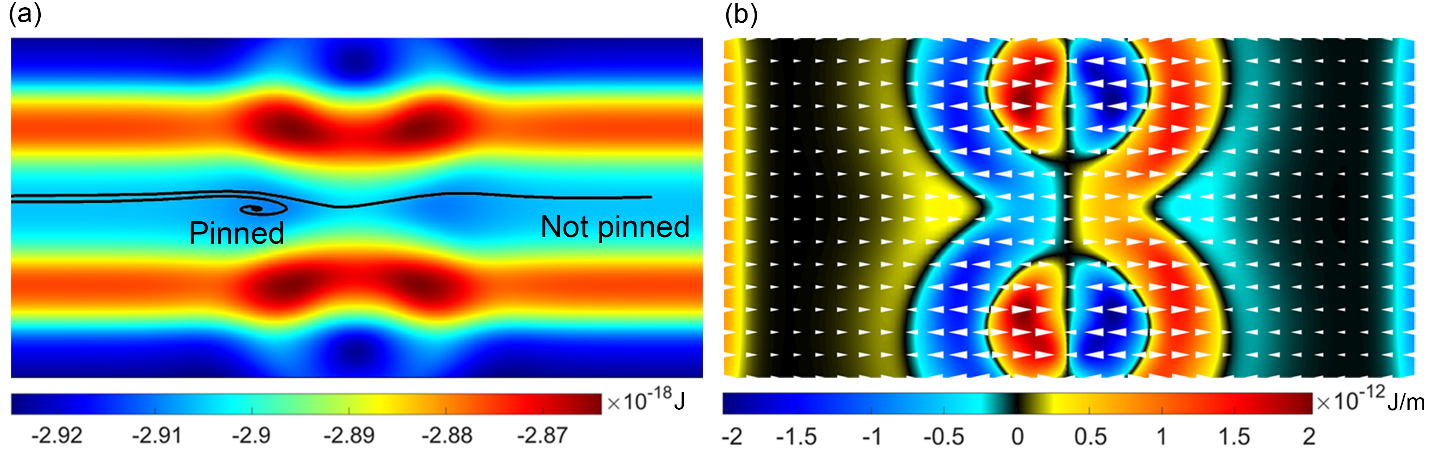


**Supplementary Figure S2.** (a) 2D plot of total energy of the system as a function of skyrmion position in a nanowire with a potential barriers at both top and bottom edges. Black solid lines are trajectories of skyrmions driven from left to right by low current density pinned at the pinning potential and driven by high current density not pinned by the pinning potential. (b) 2D plot of force acting in the positive *x*-direction on a skyrmion travelling in the *x*-direction as a function of skyrmion position in the nanowire. The white arrows show the direction of force acting on the skyrmion and scales with the strength, forces on the left and right edge originate from skyrmion-edge repulsion.

**Skyrmion Evolution under an External Stimuli**

To study the effects of varying *K_U_* and *B_z_* on skyrmions and the evolution of skyrmions under such external excitations, skyrmions are nucleated in thin films of different geometries, nanodisk, nanowire and curbed nanowire similar to that used in our proposed structure. *K_U_* and *B_z_* are then varied individually varied to study the skyrmion response to the external excitations. External stimuli are applied gradually with constant ramp rate and also as a series of Heaviside step function. Due to the presence of the Dzyaloshinskii-Moriya interaction in the material, skyrmion breathing modes are observed to be excited and can be seen more clearly when external stimuli are applied as Heaviside step function. The modal frequencies are higher when the skyrmion is compressed to smaller sizes due to external stimuli and lower when returned to their normal size. This can be observed by comparing Figure S3 and S4 with the nanodisk and nanowire showing lower and higher frequencies respectively. On the other hand, Lower frequency breathing modes are observed when the skyrmion is in curbed nanowires as shown in Figure S5. The addition of curbs to the nanowire lowers the damping and decreases the modal frequency. This is due to the demagnetizing field emanating from the ferromagnetic curb which favors the magnetization of the skyrmion.


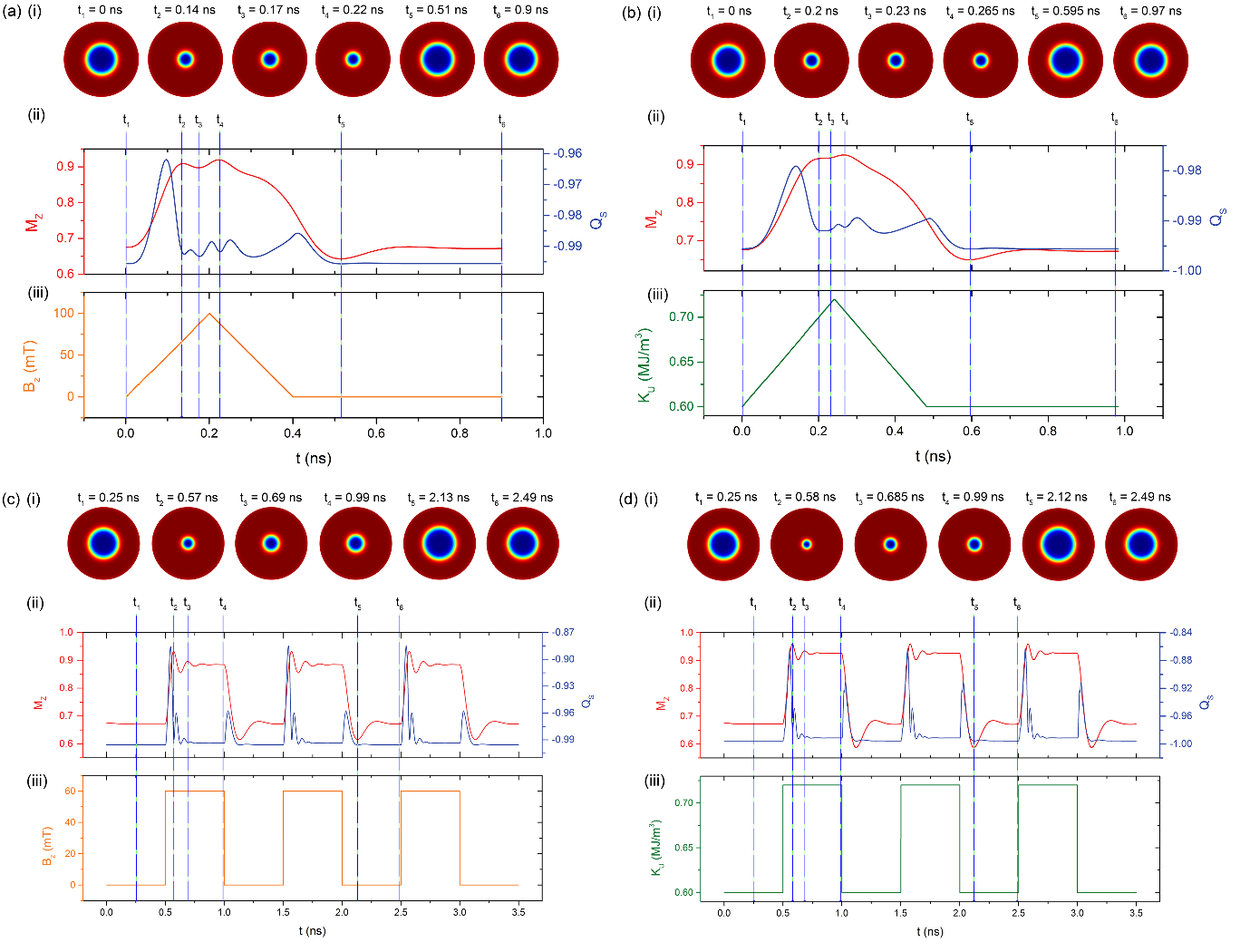


**Supplementary Figure S3.** Micromagnetic simulation of a skyrmion in a nanodisk of radius 64 nm with (a) applied magnetic field ramping from 0 to 100 mT and back to 0 at a rate of 0.5 mT/ps, (b) VCMA ramping from 0.6 MJ/m^3^ to 0.72 MJ/m^3^ and back to 0.6 MJ/m^3^ at a rate of 0.5 kJ/m^3^∙ps, (c) applied magnetic field and (d) VCMA switches as a series of Heaviside step functions. (i) Snapshots of simulation at different times and the relation between (ii) M_Z_ component, topological charge density and (iii) field strength as a function of time. Vertical dashed lines correspond to the times of the snapshots in (i).


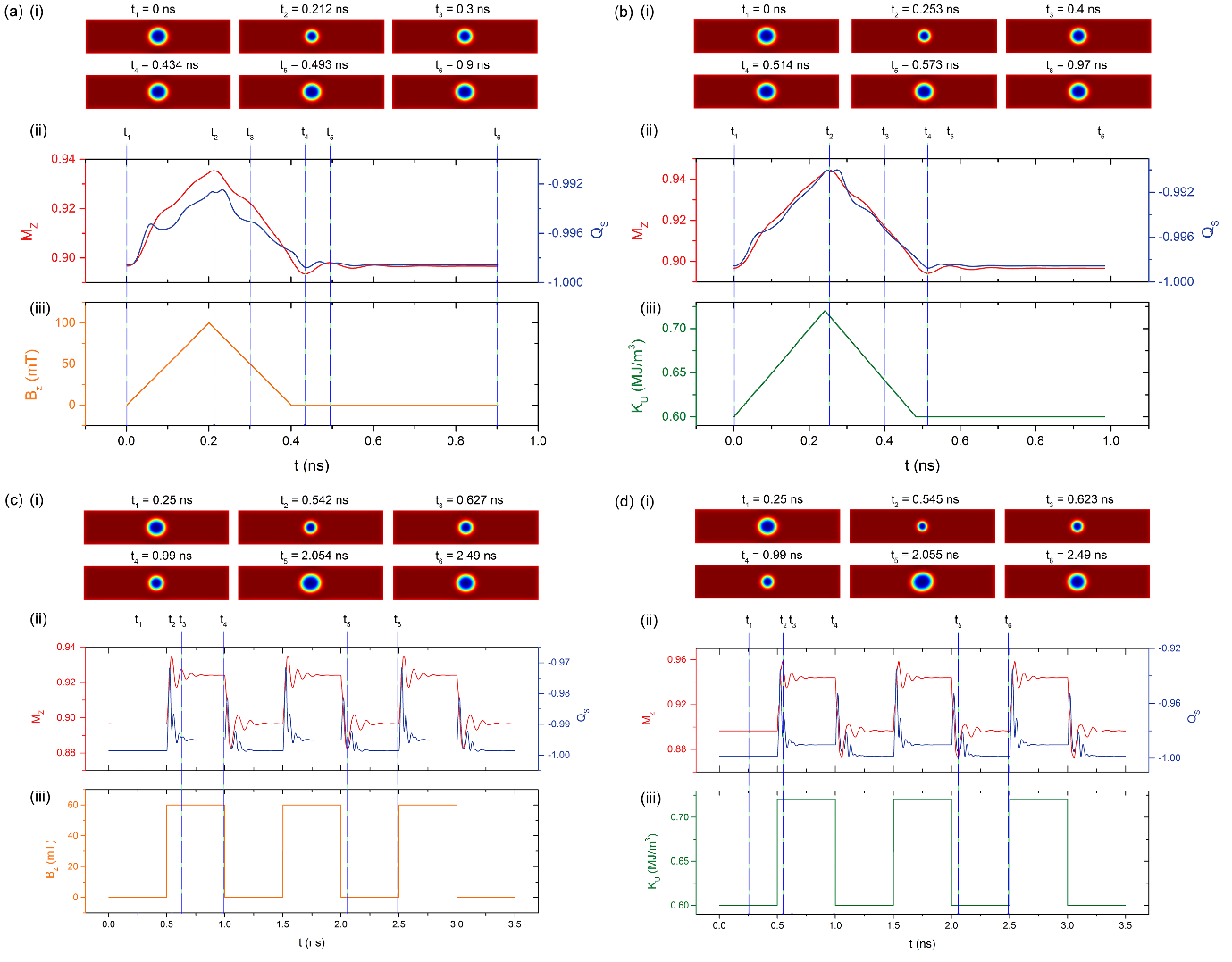


**Figure S4.** Micromagnetic simulation of a skyrmion in a nanowire of width 60 nm with (a) applied magnetic field ramping from 0 to 100 mT and back to 0 at a rate of 0.5 mT/ps, (b) VCMA ramping from 0.6 MJ/m^3^ to 0.72 MJ/m^3^ and back to 0.6 MJ/m^3^ at a rate of 0.5 kJ/m^3^∙ps, (c) applied magnetic field and (d) VCMA switches as a series of Heaviside step functions. (i) Snapshots of simulation at different times and the relation between (ii) M_Z_ component, topological charge density and (iii) field strength as a function of time. Vertical dashed lines correspond to the times of the snapshots in (i).


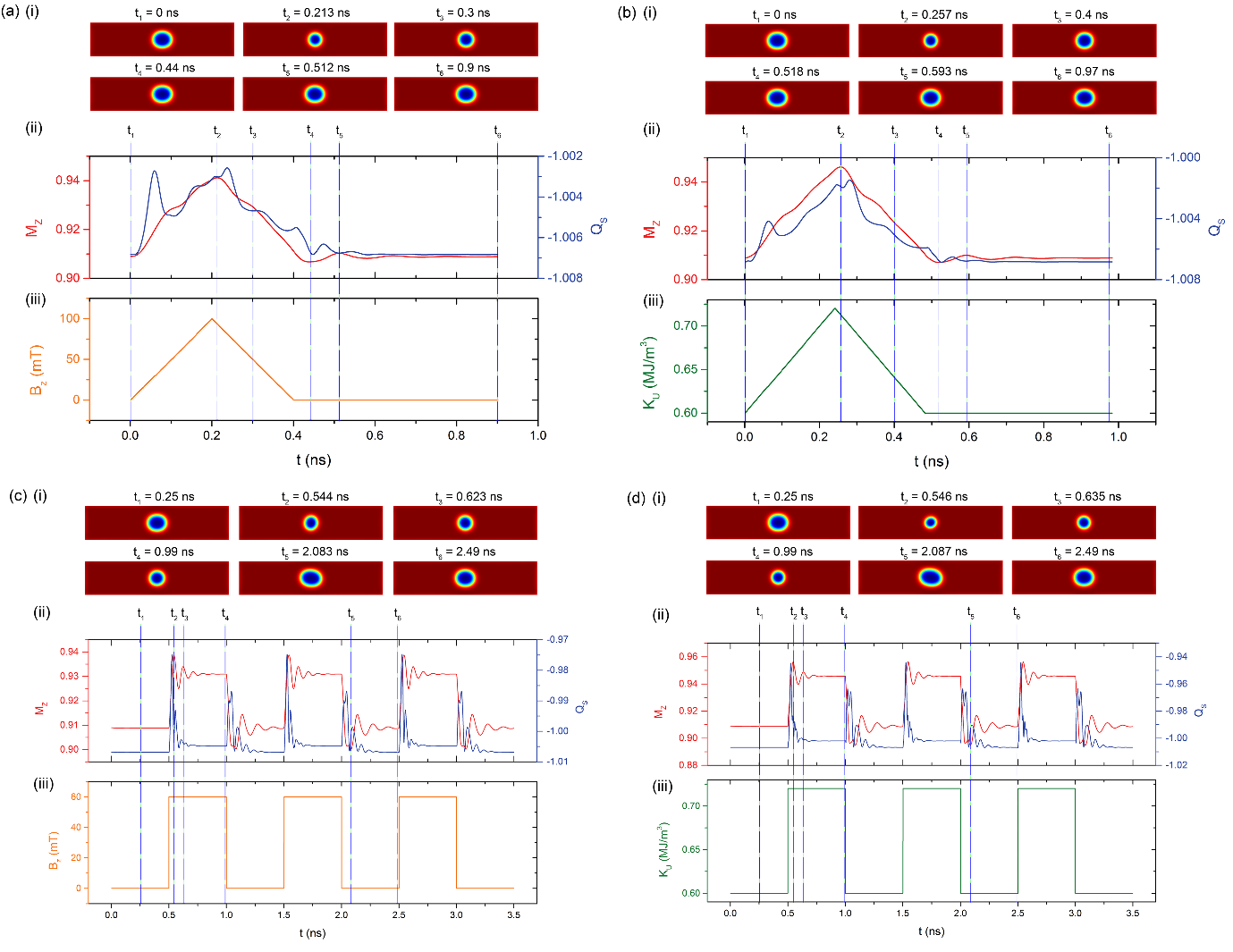


**Figure S5.** Micromagnetic simulation of a skyrmion in a nanowire of width 60 nm with 10 nm wide curbs at both edges, (a) applied magnetic field ramps from 0 to 100 mT and back to 0 at a rate of 0.5 mT/ps, (b) VCMA ramps from 0.6 MJ/m^3^ to 0.72 MJ/m^3^ and back to 0.6 MJ/m^3^ at a rate of 0.5 kJ/m^3^∙ps, (c) applied magnetic field and (d) VCMA switches as a series of Heaviside step functions. (i) Snapshots of simulation at different times and the relation between (ii) M_Z_ component, topological charge density and (iii) field strength as a function of time. Vertical dashed lines correspond to the times of the snapshots in (i).
